# 431. Performance of an Expert Recommendation Framework for Blood Culture Stewardship: Comparing Clinician Manual Review and Large Language Model Automation

**DOI:** 10.1093/ofid/ofaf695.144

**Published:** 2026-01-11

**Authors:** Nicholas P Marshall, Fatemeh Amrollahi, Manoj Maddali, Kameron Black, Aydin Zahedivash, Fateme Nateghi Haredasht, Stephen Ma, Amy Chang, Stan Deresinski, Niaz Banaei, Mary Kane Goldstein, Steven Asch, Jonathan H Chen

**Affiliations:** Stanford University, Palo Alto, CA; Stanford University, Palo Alto, CA; Stanford University, Palo Alto, CA; Stanford Medicine | Children's Health, Palo Alto, California; Stanford Medicine | Children's Health, Palo Alto, California; Stanford University, Palo Alto, CA; Stanford, Palo Alto, California; Stanford University School of Medicine, Stanford, CA; Stanford Health Care, Stanford, CA; Stanford University School of Medicine, Stanford, CA; Stanford University, Palo Alto, CA; Stanford University, Palo Alto, CA; Stanford University, Palo Alto, CA

## Abstract

**Background:**

The 2024 blood culture bottle shortage created an urgent need to conserve supplies and prioritize high-yield testing. Institutions turned to expert frameworks like Fabre et al. (2020), which stratify bacteremia risk by clinical presentation, though these frameworks have not been evaluated at scale. In our pilot, unguided LLM queries produced high sensitivity but poor specificity, consistent with prior literature, suggesting a tendency to overestimate infection risk. To address this, we anchored both clinician and LLM classification in the Fabre framework to improve precision and enable scalable clinical decision support.

**Figure 1:**
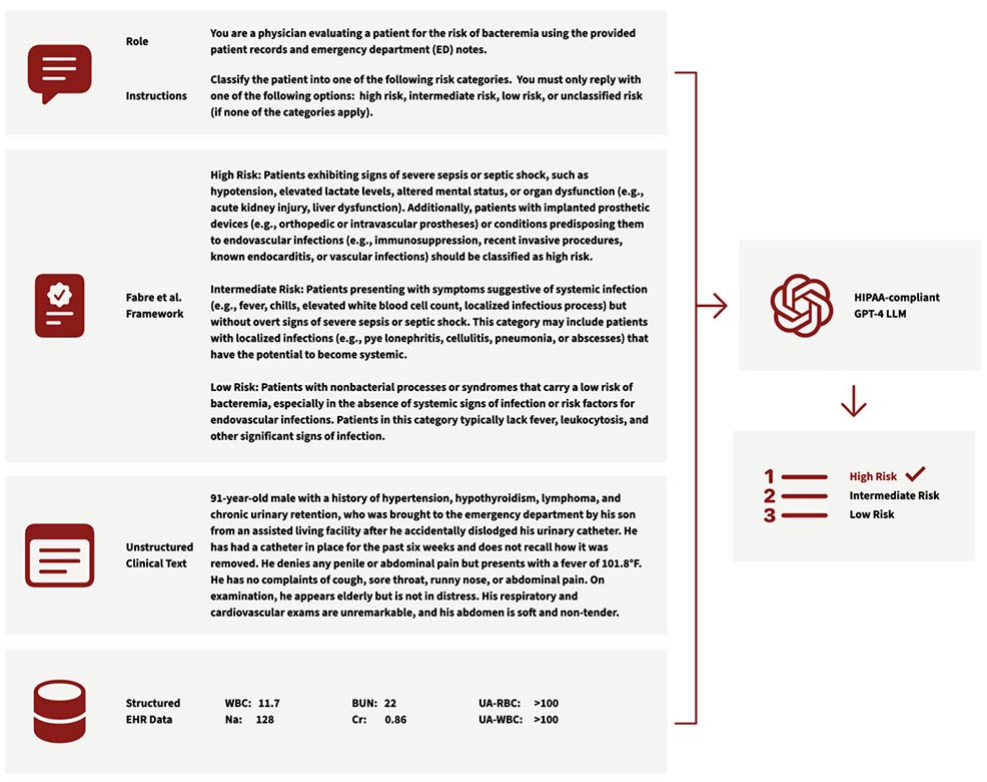
Large Language Model (LLM)-Based Pipeline for Automated Risk Stratification of Bacteremia

Schematic diagram illustrating the structured pipeline leveraging a HIPAA-compliant GPT-4 model to automate bacteremia risk assessment. The pipeline integrates the expert recommendation framework (Fabre et al., 2020) within a structured, prompt-based evaluation. Inputs to the LLM include the expert recommendation criteria, structured electronic health record (EHR) patient data, and emergency department (ED) provider notes recorded at the time of blood culture ordering. The figure also details the complete structure of prompts used to generate risk stratification outputs.

**Table 1:**
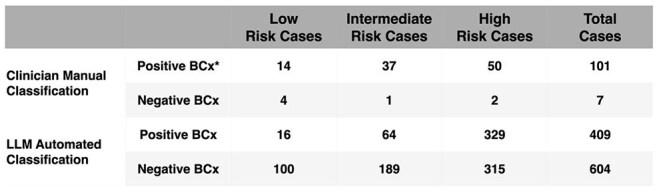
Case Stratification by Clinician Manual vs. Large Language Model (LLM) Automated Classification * BCx = blood cultures. This table shows the distribution of reviewed emergency department (ED) patient cases stratified by bacteremia risk categories using clinician manual classification (n = 108) and automated LLM-based classification (n = 1,013). Risk categories reflect pre-test probability for bacteremia as defined by the expert recommendation framework, for example:

- Low Risk: Isolated fever and/or leukocytosis, uncomplicated lower urinary tract infection, non-severe cellulitis.

- Intermediate Risk: Acute pyelonephritis, cholangitis, severe community-acquired pneumonia (CAP).

- High Risk: Catheter-associated bloodstream infection, meningitis, diskitis/native vertebral osteomyelitis.

**Methods:**

We conducted a two-phase evaluation. First, four physicians independently reviewed 108 emergency department (ED) cases with blood culture orders (80% positive), stratifying bacteremia risk using the Fabre framework; discrepancies were resolved by an expert reviewer. Second, we automated stratification using a HIPAA-compliant GPT-4 LLM with structured prompts incorporating the framework, electronic health record (EHR) data, and ED provider notes. For both approaches, low-risk classifications were treated as predicted negatives; intermediate/high-risk as predicted positives. LLM evaluation included 1,013 patient-encounter-orders, with intentional oversampling of positives (prevalence 19%).

**Table 2:**
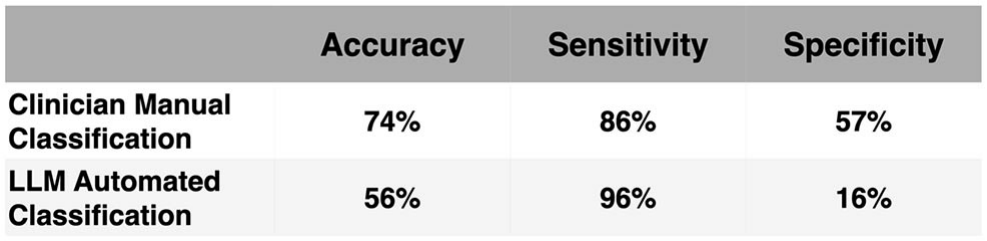
Comparison of Performance Metrics for Clinician Manual vs. Large Language Model (LLM) Automated Classification

Comparative performance of clinician manual classification and automated LLM-based classification for predicting bacteremia risk in ED patients. Metrics reported include accuracy (i.e., proportion of cases correctly classified), sensitivity (i.e., ability to correctly identify patients with true positive blood cultures), and specificity (i.e., ability to correctly identify patients with negative blood cultures).

**Results:**

Manual review achieved 86% sensitivity and 57% specificity, missing 14 positive cultures and recommending cultures in 3 negatives. LLM classification yielded 96% sensitivity and 16% specificity, correctly identifying 393 of 409 true positives but over-classifying 315 of 591 negatives.

**Conclusion:**

The Fabre framework offers structured guidance, but manual application showed limited performance and is impractical at scale. LLM-based automation enabled large-scale stratification but lacked specificity, producing excess false positives. A hybrid model, using the LLM to exclude low-risk cases and refer higher-risk cases for clinician review, may improve accuracy and resource use, highlighting the role of generative AI in scaling framework validation.

**Disclosures:**

All Authors: No reported disclosures

